# Growth of rehabilitation students in first and second years: A text mining approach

**DOI:** 10.20407/fmj.2024-024

**Published:** 2024-12-27

**Authors:** Yin Xu, Hirofumi Ota, Soichiro Koyama, Shigeo Tanabe, Kazuya Takeda, Shota Suzumura, Hiroaki Sakurai, Yoshikiyo Kanada

**Affiliations:** 1 Graduate School of Health Sciences, Fujita Health University, Toyoake, Aichi, Japan; 2 Faculty of Rehabilitation, Fujita Health University, School of Health Sciences, Toyoake, Aichi, Japan

**Keywords:** Rehabilitation, Student growth, Text mining, Professional identity

## Abstract

**Objectives:**

This research used text mining to determine the impact of curricular experiences in each year of study on the formation of professional identity among students aspiring to become physical therapists (PTs) and occupational therapists (OTs).

**Methods:**

This study included 210 students (126 PT and 84 OT) enrolled at a single rehabilitation university in Japan in 2020 and 2021. These participants completed an open-ended questionnaire on personal growth 6, 12, 18 and 24 months after enrollment. Text mining was used to extract frequently occurring nouns from descriptive text data at these four time points. A hierarchical cluster analysis was then performed to generate clusters. The number of students mentioning at least one noun in each cluster was counted, and the proportion of students in each cluster was compared across the four time periods using Cochran’s Q test.

**Results:**

The 16 nouns that appeared more than 30 times in 1073 sentences were classified into four clusters: “action plan for passing the credit certification examination”, “communication skill”, “medical knowledge” and “school life with clinical practice in mind”. The proportion of students belonging to the four clusters varied across periods. “Action plan for passing the credit certification examination” and “communication skill” differed significantly across periods (p<0.0001 and p=0.0008, respectively).

**Conclusions:**

The results reveal how students have grown in their curriculum. Their growth was transformative.

## Introduction

Medical education requires the development of a professional identity, and medical students form their identity through clinical practice and other activities.^1,2^ In nursing education, professional learning through clinical practice and lectures is seen as essential for identity formation.^3^ Physical therapists (PTs) and occupational therapists (OTs) are rehabilitation professionals who primarily improve functional impairments and activity limitations and promote social participation.^4,5^ Therefore, educators also need to foster professional identity among rehabilitation students.^6^

Reflective writing is an effective pedagogy for identity formation. Medical education provides students with opportunities to reflect on personal experiences to develop and maintain a beneficial curiosity and introspective self-awareness.^7^ It is argued that reflection requires the ability to examine, interpret and understand experiences to guide future actions^8^ and that new identities are acquired through this process.^9^ Furthermore, the reliability and validity of using reflective writing to assess the achievement of reflective skills^10^ and as an indicator of identity development has been tested.^11^ Reflective writing is also used in the education of PTs and OTs. Williams et al. analyzed the written reflections of 58 physical therapy students who recorded their perceptions of how they were adjusting to problem-solving education.^12^ Chung et al. used reflective writing to determine the impact of active learning on occupational therapy student’s autonomy and problem-solving skills.^13^ These studies both aimed to test the effectiveness of teaching methods by assessing target students’ written reflections on their experiences of a limited number of subjects and specific teaching methods in the curriculum. Other studies have compared rehabilitation students’ written impressions of their profession at the beginning and end of the course.^14^ However, in such studies, reflective writing is only recorded at two points in time – entry and graduation.

First- and second-year students acquire the knowledge necessary to become PTs and OTs through lectures. First-year students learn interpersonal skills such as communication, acquiring more specialized knowledge as they move up through the grades. In the second year of study, students experience long-term clinical practice for the first time. Therefore, although first- and second-year students are at a crucial juncture for identity formation, it is unclear how they grow and change as they experience the curriculum at each grade level.

If educators do not quantitatively examine the concepts presented in reflective writing, they may make false assumptions about the quality of student experiences.^15,16^ To solve this problem, text mining is used to quantitatively analyze reflective writing in medical education. For example, text mining has been used to quantitatively analyze reflective writing that describes medical students’ clinical practice experiences in community medicine,^17^ outpatient and home visit care^18^ and emergency medicine.^19^ The results of these analyses were derived from relationships between words that occurred frequently in the students’ reflective writings. In a rehabilitation-related study, Kitamura et al. used text mining to identify changes in the goals of prospective PT and OT students from the time they entered school.^20^

The curriculum of rehabilitation universities in Japan is organized based on the curricula and regulations for PT and OT schools established by the Ministry of Health, Labour, and Welfare (Designated Rules). In addition, the Designated Rules divide subjects into basic, special-basic, and special fields. Most universities develop their curricula in this manner, with a minimum of 800 hours of clinical practice required in special fields. Long-term clinical practice often begins in the second year, with more time devoted to it in the third and fourth years.

The purpose of this study was to use a text mining approach to determine the impact of first- and second-year curricular experiences at each grade level of a single Japanese rehabilitation university on the professional identity formation of students who aspire to become PTs and OTs. We analyzed the reflective writing performed in the first and second years, evaluating the growth that students reported experiencing, initially through lectures, and then from undertaking clinical practice for the first time. Additionally, we analyzed reflective writing from lecture learning to clinical practice longitudinally to examine changes in growth. We hypothesized that, in the first year, students would acquire interpersonal skills as they become accustomed to university life, and in the second year, they would acquire professional knowledge and social communication skills as they learn specialized knowledge and experience conversations with patients and therapists in clinical practice.

## Methods

### Participants

A total of 233 students enrolled at a single rehabilitation university in Japan in 2020 and 2021 were recruited. After excluding those who repeated the same class, 210 were included in the analysis. Specifically, 126 PT students (60 in 2020 and 66 in 2021) and 84 OT students (41 in 2020 and 43 in 2021) participated. This study was approved by the Fujita Health University Research Ethics Review Committee (HM21-377).

### Procedure for reflective writing

**Figure 1:**
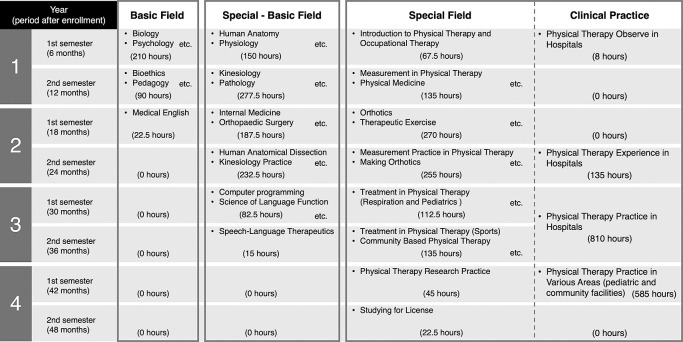
Study content for students enrolled in 2020 and 2021 at the X University School of Health Sciences Faculty of Rehabilitation, Major of Physical Therapy

**Figure 2:**
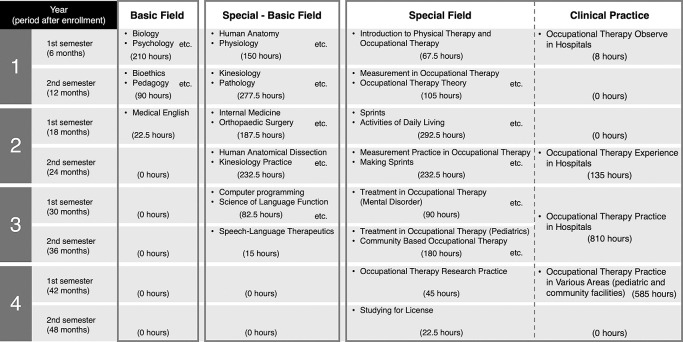
Study content for students enrolled in 2020 and 2021 at the X University School of Health Sciences Faculty of Rehabilitation, Major of Occupational Therapy

**Figure 3:**
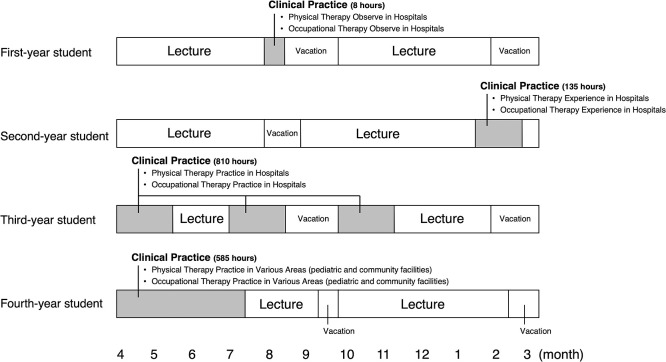
Timing and content of clinical practice at X University

In the curriculum at X University, students take a basic field in the first semester of their first year, a basic-special field in the second semester of their first year, and a special field between the second and fourth years. Although PT and OT are different healthcare professions, students receive similar health professional education in the basic and basic-special fields. X University requires a minimum of 1500 hours of clinical practice experience. Long-term clinical practice begins in the second semester of the second year (135 hours). Time devoted to clinical practice in the third (810 hours) and fourth (585 hours) years is longer than that at any other Japanese university (Figures 1, 2 and 3). All participants took the first- and second-year curriculum at X University. Participants completed reflective writing four times after the curriculum began (at 6 months [at the end of the first semester of the first year], 12 months [at the end of the second semester of the first year], 18 months [at the end of the first semester of the second year], and 24 months [at the end of the second semester of the second year]). For reflective writing, the students used “Assessmentor” (Learning and Development System Design Laboratory, Inc., Hyogo, Japan), a learning outcomes visualization system with which they could regularly self-evaluate their goals and achievements.^21^ The study participants were asked the following four questions:

Q1 (6 months after enrollment): The first semester is over. How do you think you have grown?

Q2 (12 months after enrollment): How have you grown since you first enrolled?

Q3  (18 months after enrollment): The first semester of the second year is completed. How have you grown?

Q4 (24 months after enrollment): Half of your university life is over. How have you grown?

The purpose of the questionnaire and use of the assessor were explained to the participants in advance. Participants used their tablets and had approximately 20 min to answer the questions.

### Data analysis

Text mining and hierarchical cluster analysis were performed on the textual data collected using procedures as reported in previous studies.^22,23^ We compiled the growth data for all periods using text mining; text mining was performed using the KH Coder (version 3, Koichi Higuchi, Kyoto, Japan) software. We corrected typographical, spelling and punctuation errors in the data in preparation. Synonyms were merged into a single word while retaining the meaning of the text. Nouns were extracted using the KH Coder. We excluded the words “physical”, “occupational”, and “therapy” because of their potential to extract content specific to each profession. The words “growth” and “oneself” were excluded because they do not relate to the specifics of growth. For hierarchical cluster analysis, only nouns that appeared more than 30 times during the entire period were selected. We classified the nouns into clusters using the agglomeration dissimilarity coefficient based on the Jaccard distance as a measure of co-occurrence for term pairs, and the resultant hierarchical cluster analysis dendrogram was located using the KH Coder. The smaller the value of the agglomeration dissimilarity coefficient, the higher the co-occurrence and the stronger the word association. The threshold for the agglomeration dissimilarity coefficient was set at the lowest co-occurrence between clusters obtained in the cluster agglomeration process, confirmed by the authors (YX, HO, SK, ST, SS, HS).

To examine changes in participants’ growth, we counted the number of students who wrote sentences containing at least one noun from each cluster in each period. For statistical analysis, we used Cochran’s Q test to compare the proportion of students within each cluster. McNemar’s test was used to compare the four periods within each cluster, and the level of statistical significance using Bonferroni correction was 0.05/6=0.0083. We used SPSS (version 28, IBM Corp., Armonk, NY) for statistical analyses.

## Results

**Figure 4:**
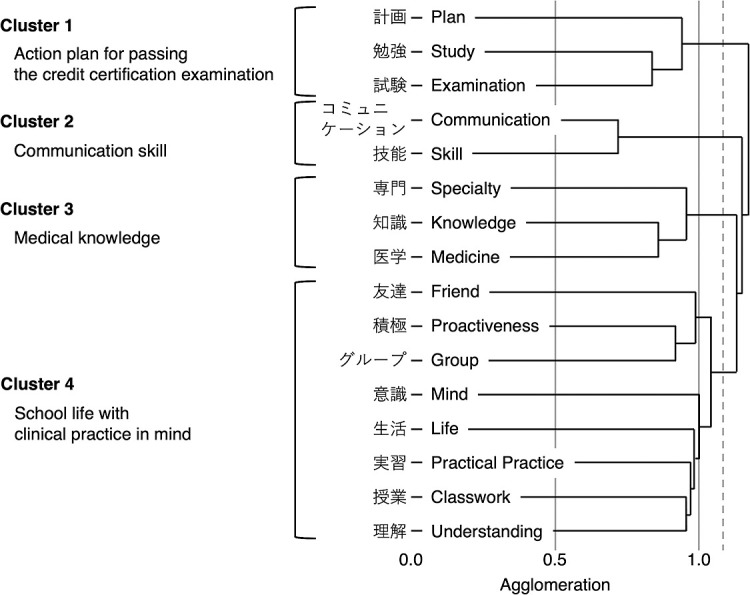
Dendrogram of clusters Nouns extracted from the text data of students’ answers across all time periods were classified into four clusters: “Action plan for passing the credit certification examination”, “Communication skill”, “Medical knowledge” and “School life with clinical practice in mind”. The dotted vertical line represents the threshold of the agglomeration dissimilarity coefficient (approximately 1.1), which determines the number of clusters.

A total of 210 students wrote 1073 sentences. These included 1292 morphemes and consisted of 17,427 words. Among these, 490 nouns appeared 2666 times. A hierarchical cluster analysis was performed using 16 nouns that appeared more than 30 times. In the hierarchical cluster analysis, agglomeration dissimilarity was more than twice as high when the number of clusters was four. The authors, therefore, classified these nouns into four clusters. The clusters were named “action plan for passing the credit certification examination”, “communication skill”, “medical knowledge” and “school life with clinical practice in mind” (Figure 4).

**Figure 5:**
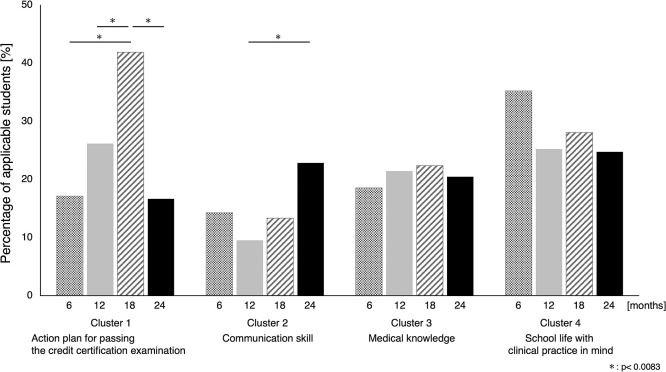
Percentage of students answering the words in each cluster

Across time points, the percentage of participants who wrote the nouns that make up each cluster was the highest at 41.9% for “action plan for passing the credit certification examination” at the 18-month reflection and below 40% for other cases. Cochran’s Q test revealed a significant difference in “action plan for passing the credit certification examination” (p<0.0001) and “communication skill” (p=0.0008). The percentage of respondents whose answers included “action plan for passing the credit certification examination” increased between 6 months and 18 months and then decreased at 24 months—the proportion of participants writing nouns in this cluster was significantly higher at 18 months than that at other time periods. “Communication skill” was included in a higher percentage of respondents’ writings at 24 months than at the other points. A significant difference was observed between 12 and 24 months. The proportion of students writing nouns from the “medical knowledge” cluster in each period ranged from 18.6% to 22.4%, with little difference between them. The percentage of participants in each period that included “school life with clinical practice in mind” in their writing ranged from 24.8% to 35.2% (Figure 5, Table 1).

## Discussion

In this study, we used text mining to analyze the growth of first- and second-year PT and OT students over four semesters using textual data from their 6-monthly reflective writing exercises. As a result, their growth was categorized into four clusters: “action plan for passing the credit certification examination”, “communication skill”, “medical knowledge”, and “school life with clinical practice in mind.” The proportion of participants writing the nouns that composed each cluster changed as the curriculum progressed.

Medical and nursing schools worldwide have introduced reflective writing as part of education.^10,24^ However, when evaluating reflective writing, qualitative judgments can be ambiguous owing to reader bias; therefore, quantitative analysis is necessary.^25^ Text mining can extract patterns and relationships in data from large data sets, enabling deeper analysis and knowledge discovery. Recently, text mining has been applied to large student education datasets to interpret academic performance.^26^ In this study, we used this approach to quantitatively analyze the textual data of student reflections. This allowed us to quantitatively analyze student growth during each period.

The number of students whose writing included nouns in the “action plan for passing the credit certification examination” cluster increased from 36 to 88 over the period from 6 to 18 months. This could be explained by student anxiety about exams. About half of rehabilitation students studying professional knowledge and skills present with anxiety symptoms.^27^ Research has shown that anxiety is associated with lower academic performance.^28^ Because the first- and second-year students were learning specialized knowledge and skills for the first time, it was assumed that many of them had high levels of anxiety. To alleviate anxiety and increase performance, the students in this study could have made action plans with the goal of passing the credit examination. In addition, the curriculum for the first semester of the first year (6-month time point) at X University was 49.1% basic field, 35.1% basic-special field and 15.8% special field; for the second semester (12 months), 17.6% basic field, 54.4% basic-special field and 28.0% special field; and for the first semester of the second year (18 months) 4.7% basic field, 39.1% basic-special field and 56.3% special field. Thus, students had spent a relatively long time studying the basic field for the 6 months, basic-special field for the 12 months, and special field for the 18 months time points. Therefore, opportunities to acquire professional knowledge and skills gradually increased, with students recognizing that these are necessary to become PTs and OTs and that planning is necessary to acquire them.

The highest number of students whose answers reflected “communication skill” was at 24 months. The students received 135 hours of clinical practice over several weeks between 18 and 24 months. According to the World Confederation for Physical Therapy, clinical students are expected to be progressively exposed to patients with various diagnoses. In addition, PT training is expected to include timely and constructive feedback on professional skills and clinical reasoning.^29^ We believe that the clinical practice experienced in this study provided an opportunity for students to communicate with patients, rehearse explanations and provide feedback regarding assessment and training. We also considered that it provided the opportunity to communicate with mentors to receive feedback on those skills. This first experience of clinical practice may have made many students aware of the importance of their communication skills. Furthermore, it has been shown that sufficient patient interaction is necessary for students to better form their professional identity.^1^ The students in this study are expected to further develop their professional identity as PT and OT through their experience of interacting with patients during lengthy clinical practice in their third and fourth years.

The percentages of students whose answers involved “medical knowledge” and “school life with clinical practice in mind” were not significantly different across all periods. However, the number of students whose writing included “medical knowledge” increased slightly between 6 and 18 months. This may be because of increased opportunities to acquire specialized knowledge and skills. The highest number of students who reflected on “school life with clinical practice in mind” was at 6 months. Kitamura et al. used text mining to analyze the goals of first-year PT and OT students at the time of their enrollment and 6 and 12 months later. They found that the number of students who answered “Clinical Practice” gradually increased.^20^ Kitamura’s thesis was completed at X University, where our participants were studying. X University is adjacent to X University Hospital, where students receive clinical practice. The students in this study were immersed in this learning environment and, thus, may have been aware of the clinical practice they would experience in the future.

Although each cluster consisted of nouns that appeared frequently in student’s textual data, fewer than half of the students answered the questions. Although the participants in this study had the same curriculum, their experiences and impressions of lectures and clinical practice were not necessarily the same. It was assumed that students’ reflections were structured based on their impressions and experiences. There were 499 different nouns in the reflection forms completed by the 210 students, possibly indicating individual differences in impressions and experiences.

Text mining in this study was used to analyze textual data from four reflective writings written over the 24 months after enrollment. The timings of these writings aligned with the curriculum of X University. Educators can assess the effectiveness of learning by comparing students’ growth with the curriculum. Furthermore, by evaluating the same student’s reflective writings at different points in time, educators can gain an understanding of their development processes. These results can be used to develop or modify curricula.

The reflections in this study were written over a duration in which students went from attending lectures at school to experiencing clinical practice at a hospital. The first long-term clinical practice that students experienced was a valuable opportunity to learn the specialized skills required for PT and OT. However, the results of this study showed that participants were more likely to act, earn credits, and make academic progress towards becoming PT and OT during the 6–18 months before clinical practice. Lectures at school represented an opportunity to learn professional knowledge. Therefore, the results of this study suggest that students developed more effective learning strategies as they advanced from the first to the second year.

This study has some limitations. First, the outcome of this study was the growth students experienced while engaging with a single university’s curriculum. First- and second-year students at X University experienced the curriculum at each grade level and were able to identify the extent of their growth through lectures and clinical practice. However, the hours of lectures delivered over the 6–24 month period may differ from those at other universities. Similar studies conducted at other universities could facilitate the comparison of results to assess the impact of different curricula. Second, the questionnaires used in this study did not determine the range of the reflective period. The students participating in this study may have reflected on growth occurring within the specific periods mentioned in the questionnaire or across the entire time since their enrollment. Educators need to clarify their questions according to what they want to learn about student growth, a new challenge that this study helped us identify. Third, only a few students mentioned goals in their responses to the questions used in this study. Therefore, they were included in the data analysis, although not enough to distort the results of this study. In future studies, the analysis method needs to be improved so that only information about growth is extracted.

The study population comprised only rehabilitation students. Students aiming to become other types of medical professionals also learn their professional identities through lectures and clinical practice. However, such students may not necessarily consider their own growth, as shown by the results of this study. Future research could examine these students together, shedding light on the developmental growth process of aspiring medical professionals.

## Figures and Tables

**Table1:** Comparison across four time periods for each cluster

Cluster	Cochran’s Q test p-value	McNemar’s test standardized statistic p-value					
		6 vs. 12 months	6 vs. 18 months	6 vs. 24 months	12 vs. 18 months	12 vs. 24 months	18 vs. 24 months
Action plan for passing the credit certification examination	<0.0001	0.0256	<0.0001	1.0000	0.0009	0.0175	<0.0001
Communication skill	0.0008	0.1547	0.8852	0.0282	0.2561	0.0004	0.0097
Medical knowledge	0.7356	0.4962	0.3740	0.6774	0.8937	0.8973	0.6583
School life with clinical practice in mind	0.0506	0.0340	0.1378	0.0174	0.5663	1.0000	0.4825

Cochran’s Q test p-value: <0.0500 McNemar’s test standardized statistic p-value: <0.0083 (Bonferroni correction: 0.05/6)
